# New Medical College in Philadelphia

**Published:** 1881-04

**Authors:** 


NEW MEDICAL COLLEGE IN PHILADELPHIA.—Whether a *necessity* for another medical college exists in Philadelphia, or not, a new one has been organized there, so we learn from the medical press of that city. One thing can be said in behalf of the enterprise, viz.: it proposes an increase of time in the school, beyond what most of the schools pursue, before the student is eligible for graduation. The feature of a free course on English science and literature, and the elements of Latin, for such students as have not enjoyed a classical education, is also a commendable one. It is a mistake to suppose that the movement toward a higher and more thorough preparation for the doctorate is waning, because Bellevue has fallen back from her high resolve, and some other schools present a weakening of their spinal columns!

